# Time dependent decomposition of ammonia borane for the controlled production of 2D hexagonal boron nitride

**DOI:** 10.1038/s41598-017-14663-8

**Published:** 2017-10-30

**Authors:** Vitaliy Babenko, George Lane, Antal A. Koos, Adrian T. Murdock, Karwei So, Jude Britton, Seyyed Shayan Meysami, Jonathan Moffat, Nicole Grobert

**Affiliations:** 1 Department of Materials, University of Oxford, Oxford, OX1 3PH UK; 2 Oxford Instruments Asylum Research, High Wycombe, HP12 3SE UK; 3 Williams Advanced Engineering, Grove Oxfordshire, OX12 0DQ UK; 4 Present Address: Centre for Advanced Photonics and Electronics, University of Cambridge, 9 JJ Thomson Ave, Cambridge, CB3 0FA UK; 5 Present Address: Department of Chemistry, University of Cambridge, Lensfield Rd, Cambridge, CB2 1EW UK; 6 Present Address: Nanostructures Department, Institute of Technical Physics and Materials Science, Centre for Energy Research, PO Box 49, H-1525 Budapest, Hungary; 7 Present Address: CSIRO Manufacturing, P.O. Box 218, Bradfield Road, Lindfield, New South Wales 2070 Australia; 8 Present Address: Renishaw New Mills, Wotton-under-Edge, Gloucestershire, GL12 8JR UK

## Abstract

Ammonia borane (AB) is among the most promising precursors for the large-scale synthesis of hexagonal boron nitride (h-BN) by chemical vapour deposition (CVD). Its non-toxic and non-flammable properties make AB particularly attractive for industry. AB decomposition under CVD conditions, however, is complex and hence has hindered tailored h-BN production and its exploitation. To overcome this challenge, we report in-depth decomposition studies of AB under industrially safe growth conditions. *In situ* mass spectrometry revealed a time and temperature-dependent release of a plethora of N_x_B_y_-containing species and, as a result, significant changes of the N:B ratio during h-BN synthesis. Such fluctuations strongly influence the formation and morphology of 2D h-BN. By means of *in situ* gas monitoring and regulating the precursor temperature over time we achieve uniform release of volatile chemical species over many hours for the first time, paving the way towards the controlled, industrially viable production of h-BN.

## Introduction

Hexagonal boron nitride (h-BN) is a layered ceramic material with an atomic structure similar to that of graphite, but with a wide band gap of 5.7 eV^1^. Monolayer or few-layer h-BN has received considerable attention recently as a substrate for graphene and other 2D-nanomaterials. Due to the low number of charge impurities in h-BN the mobility of supported graphene improved by more than an order of magnitude^2^ compared to graphene on the commonly used silicon dioxide. Highly crystalline h-BN domains serve as a better template for the epitaxial growth of monolayer graphene^3^ and WS_2_^4^, showing good alignment of the domains. The possibility of forming heterostructures wholly from the 2D family of nanomaterials opens many exciting opportunities in the electronics industry. Radio frequency electronics^5^, nanocapacitors^6^, flexible and transparent transistors^7^ have been demonstrated using h-BN and other 2D-nanomaterials.

The synthesis of high-quality h-BN by chemical vapour deposition (CVD) has many challenges, some of which are similar to those of graphene where the right CVD parameters have to be found. But an inherent difficulty for the controlled h-BN production arises from the limited choice of suitable precursors containing both nitrogen and boron. A variety of precursors have been investigated, some of which are diborane and ammonia^8^, b-tricholoroborazine^9^, decaborane and ammonia^10^ or borazine^11–18^. For example, borazine allowed the growth of micron-sized h-BN domains on Cu at temperatures between 950–1000 °C^19^ and is one of the most commonly used precursors due to its high vapour pressure. Nevertheless, these compounds are known to be toxic, highly flammable, corrosive, unstable, or difficult to store, presenting obvious safety concerns, practical challenges and higher processing costs. For this reason, ammonia borane (AB) precursor, classed as a non-hazardous substance or mixture, has been gaining popularity as the precursor of choice for h-BN synthesis^20–27^. Often, however, AB is used in combination with high hydrogen flows, defeating the purpose of using a safe precursor.

Atmospheric pressure CVD (APCVD) is a scalable 2D method that does not require costly investment or maintenance, and is suitable for *e.g.* an open roll-to-roll process^28^. To obtain sufficient concentration of N_x_B_y_-containing species in the synthesis gas, the AB precursor must be heated to temperatures where its decomposition occurs^21^. Previous decomposition studies of AB focused on the suppression of N_x_B_y_-containing species for the use of AB in hydrogen storage applications^29–31^. In contrast to this, the CVD synthesis of h-BN requires efficient conversion of AB into volatile N_x_B_y_-containing species. Several studies on h-BN synthesis refer to thermoanalytical measurements^31–33^ of AB decomposition resulting in the release of H_2_, borazine and monomeric aminoborane, but failing to note the differences in temperatures or conditions. Additionally, such measurements are performed either in an Ar carrier gas or in vacuum. The former causes difficulties in subtracting the overlapping mass spectra background^31^, while the latter does not replicate the dynamic equilibrium in an APCVD system. With the increased need for controlled and scalable production of h-BN, identifying the precise nature of the precursor species in CVD is an urgent priority.

Here, we report a time dependent release of the chemical species that are generated from a commercially-available AB precursor at various temperatures (T_AB_) using *in situ* mass spectrometry (MS). We find that seven N and B-containing species are present in the temperature range typically used for h-BN growth at atmospheric pressure. Moreover, their time-dependent flow profiles do not correlate with each other, suggesting rather complicated reaction pathways. We also find that at higher temperatures (T_AB_ >80 °C) the time-dependent profiles show a rapid peaking and decaying behaviour (within minutes), while at lower temperatures (T_AB_: 60–80 °C) the precursor decomposition is slow and varies on the order of hours. Experimental observations of h-BN deposits from different stages of the precursor decomposition show a variation in the growth rate and shape. As such, reproducible production of high quality h-BN can be difficult to achieve unless the release of volatile species is controlled. We also report solutions for controlling the occurrence of the volatile N_x_B_y_-containing species with good accuracy by regulating the precursor temperature with time to achieve reproducibility and larger h-BN domains (~50 µm).

## Results

### Identification of the chemical species released from AB decomposition

**Figure 1:**
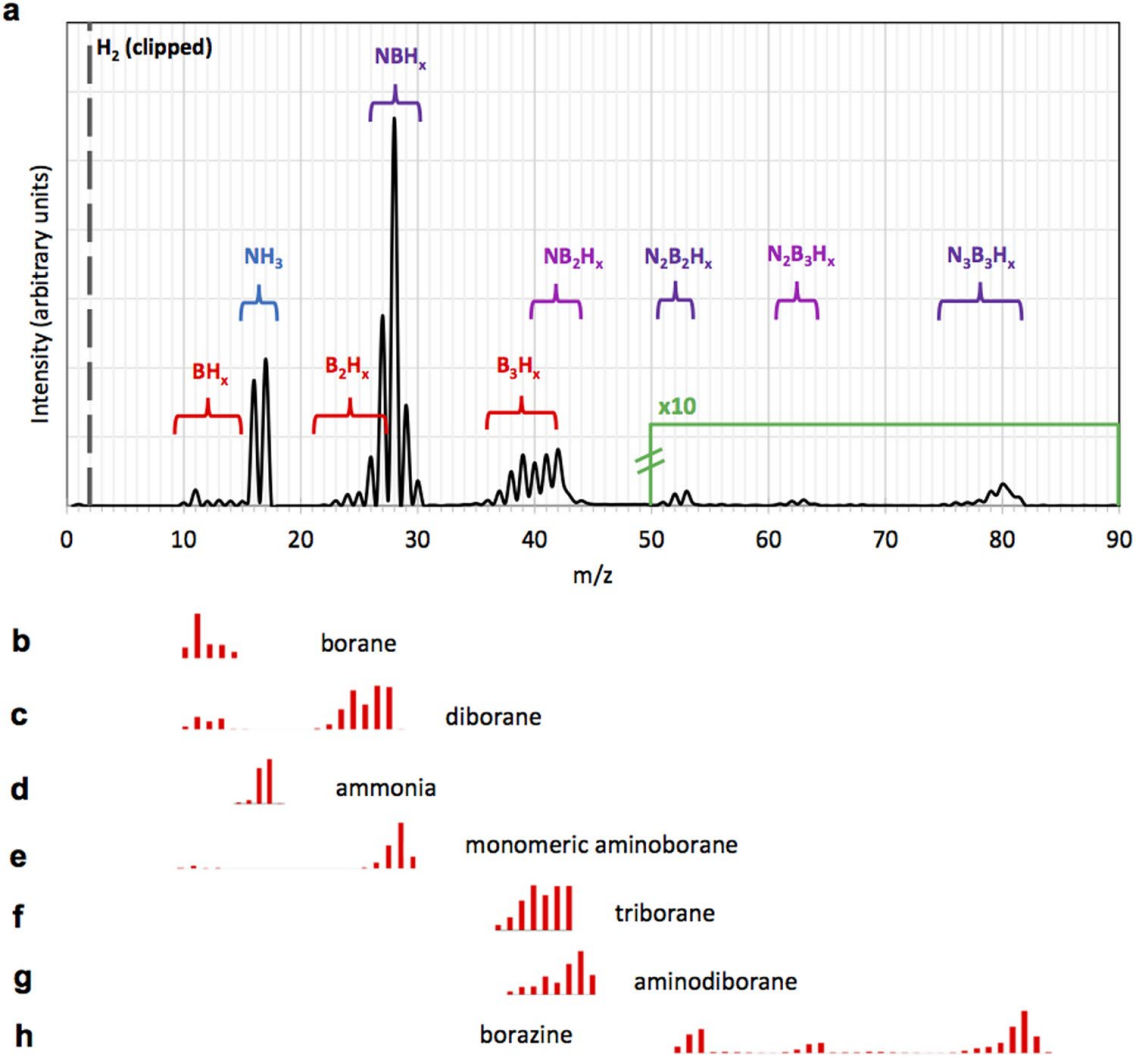
Mass spectrometry analysis of AB decomposition. (**a**) Mass spectrum of ammonia borane decomposition when heated to 100 °C in the background-free H_2_ atmosphere at ambient pressure. N_x_B_y_-containing species are assigned to different fragmentation patterns. The patterns between m/z 21–30 are deconvoluted to diborane and monomeric aminoborane; and the patterns between m/z 31–45 are deconvoluted to aminodiborane and triborane as discussed in the main text. The intensity profiles between m/z 50 and 80 (borazine) are magnified ten times. (**b**–**h**) Bar plots of the deconvoluted intensity profiles assigned to various chemical species.

The decomposition of the AB precursor was initially measured in different gas atmospheres to determine targeted information. First, the mass spectrum was recorded in pure H_2_ carrier gas to avoid the background resulting from the Ar carrier gas. The system was thoroughly purged (>12 hours) and the background was subtracted before AB was heated (T_AB_ = 100 °C). The resulting spectrum when the decomposition reaction reached a maximum is shown in Fig. 1a. AB gas (m/z 31) was not detected due to low vapour pressure at this temperature, compared to its decomposition products^31^. Some of the observed species from the plethora of fragments are commonly-known chemical compounds and their patterns can be identified. Currently there are no reports that detect all of these species simultaneously and assess their influence on h-BN synthesis. The readily identified chemical species were ammonia^34^ (NH_3_, Fig. 1d); borazine^31,35^ (N_3_B_3_H_6_, Fig. 1h), borane (BH_3,_ Fig. 1b) and diborane^34^ (B_2_H_6_, Fig. 1c). We find negligible quantity (<0.3%) of borazine in our measurements (Fig. 1a,h), in agreement with previous reports where borazine is mainly produced at higher precursor temperatures of T_AB_ > 130 °C^35^. Little identification data is available relating to the two remaining fragmentation patterns between m/z 26–30 and m/z 36–44. Monomeric aminoborane, BNH_4_, matches with the m/z 26–30 fragment and has been reported in previous studies^31,35^. The species in the m/z 36–44 range preliminary matched with convoluted patterns of hydrogen-rich triborane^36^ (B_3_H_x_) and aminodiborane^37,38^ (NB_2_H_x_). To confirm presence of these two species and to identify their fragmentation patterns an experiment was designed where the spectrum was recorded in a H_2_/NH_3_ atmosphere, such that NH_3_ was highly in excess. It is expected that excess ammonia can shift the chemical equilibrium of the reaction towards B_x_N_y_H_z_ containing species. Notably, the B_2_H_6_ and B_3_H_x_ fragmentation patterns disappeared (Supplementary Figure 1) allowing us to deconvolute them from the overlapping B_x_N_y_H_z_ species_._ Fragmentation intensities of monomeric aminoborane, triborane and aminodiborane are thus identified as shown in Fig. 1e–g respectively (Supplementary Table 1). It must be noted that no other fragments were observed at different temperatures (T_AB_: 50–110 °C), only their intensity varied depending on the temperature and time, as will be discussed later in the text.

While secondary decomposition and deposition during the transfer of molecules to the MS may be possible, it is expected to be low since the majority of the detected species are stable in the timeframe of the measurement. For example, a reaction between ammonia and diborane requires heating at 200 °C for several hours^39^ and borazine decomposes >340 °C^40^. We estimate that it takes *ca*. 30 s for the gas molecules to travel through the precursor container and the manifold to the position of the MS sampling capillary (also furnace), however, it only takes *ca*. 0.3 s to travel through the sampling capillary. Such configuration ensures the best match between the measured species and the species actually entering the reaction chamber during the synthesis. Additionally, the temperatures of the precursor chamber and the transfer manifold (including the sampling capillary) were controlled separately allowing to maintain comparable molecule flight path conditions at different precursor temperatures. The detailed CVD system configuration and the procedure for the MS decomposition measurements are described in the methods section.

### Temporal profiles of AB decomposition

**Figure 2:**
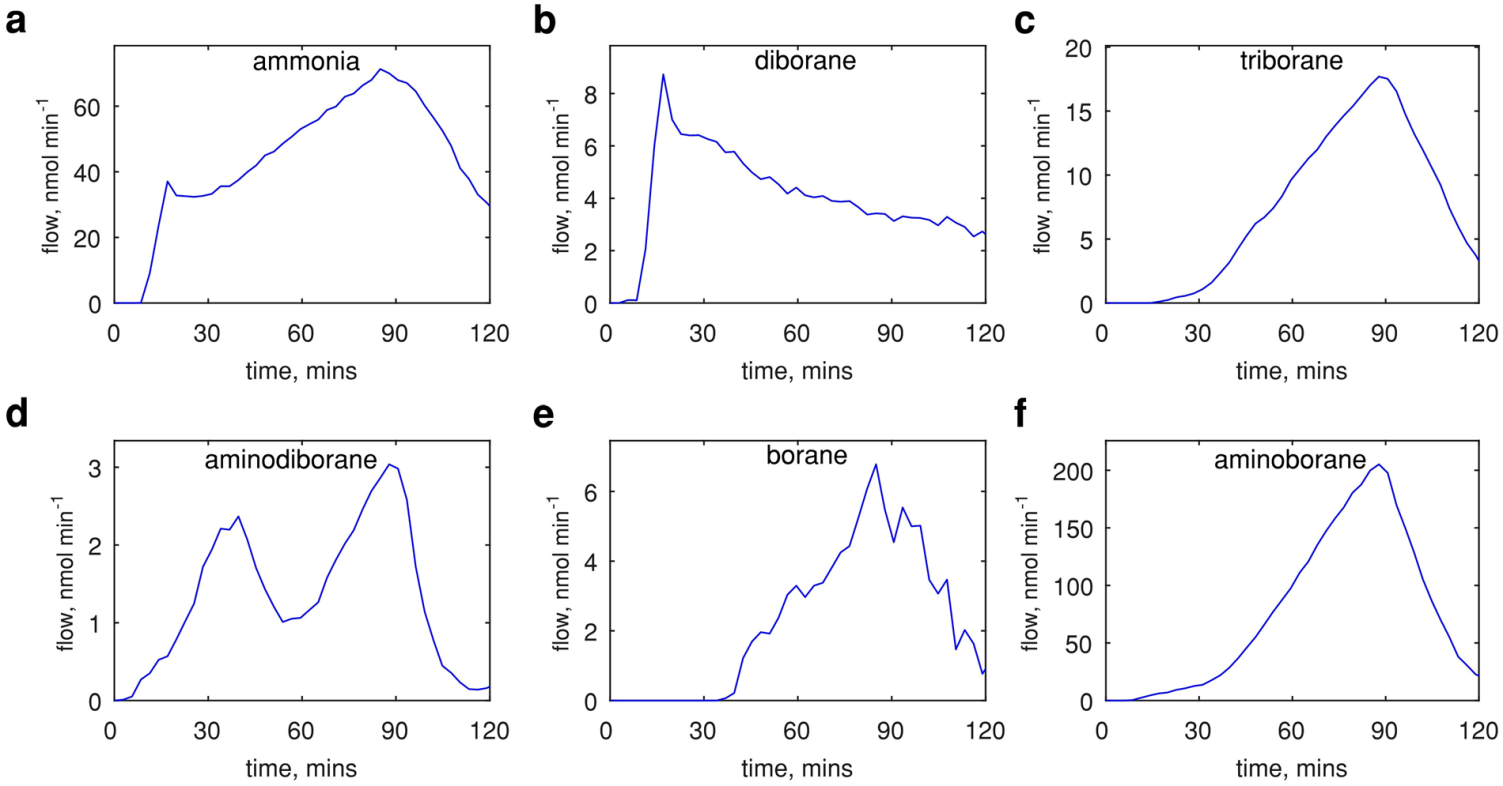
Time-dependent flow evolution profiles of individual N_x_B_y_-containing species released at T_AB_ = 90 °C. (**a**–**f**) Molar flows of ammonia, diborane, triborane, aminodiborane, borane and monomeric aminoborane respectively; showing variation throughout the time of measurement. Aminoborane becomes the dominant species at peak decomposition, while aminodiborane, borane and borazine have low contributions to the total flow.

Once the chemical species were identified in the pure H_2_ and ammonia-rich H_2_ atmospheres, follow-on measurements were performed in a non-flammable, industrially-safe 2.5 % H_2_ in Ar gas mixture to match exactly the CVD synthesis experiments for h-BN. To improve reproducibility and to avoid AB foaming, the precursor was mixed with an inert filler (methods). Exemplar time-dependent evolution profiles of all the significant N_x_B_y_-containing species released at a precursor temperature of T_AB_ = 90 °C over 2 hours are shown in Fig. 2a–f. Notably, the anticipated products of AB decomposition: NH_3_ and B_2_H_6_ appear first after around 10 minutes (including a 5 minute 50 °C pre-heating time; methods), as shown in Fig. 2a,b. The latter can be considered a product of two borane molecules that rapidly react^41^. This simple reaction pathway corresponds to the symmetrical splitting of the AB molecule,

1 $${{\rm{H}}}_{3}{\rm{N}}-{{\rm{B}}{\rm{H}}}_{3}\Rightarrow {{\rm{N}}{\rm{H}}}_{3}+{{\rm{B}}{\rm{H}}}_{3}$$

2 $${{\rm{BH}}}_{3}+{{\rm{BH}}}_{3}\Rightarrow {{\rm{B}}}_{2}{{\rm{H}}}_{6}$$

As time progresses, other species start to appear. After *ca*. 30 minutes, there is a rapid increase in their intensity, most significantly triborane and aminoborane (Fig. 2c,f). As will be shown later in the text the profiles measured at different temperatures also displayed two such stages. At lower temperatures (T_AB_ ≤ 90 °C) these stages could be deconvoluted in time, whereas at higher temperatures the transition took place very quickly. These observations suggest that the precursor changes in the duration of the measurement. Previous studies^42^ showed that AB indeed undergoes a solid-state phase change to its ionic isomer, diammoniate of diborane (DADB), albeit in different experimental conditions. This phase is more mobile^42^, explaining the observed increase in total flow of N_x_B_y_-containing species. The following chemical reaction describes this solid-state phase change:

3 $$2{{\rm{H}}}_{3}{\rm{N}}-{{\rm{B}}{\rm{H}}}_{3}\Rightarrow {[{{\rm{B}}{\rm{H}}}_{2}{({{\rm{N}}{\rm{H}}}_{3})}_{2}]}^{+}{[{{\rm{B}}{\rm{H}}}_{4}]}^{-}$$

**Figure 3:**
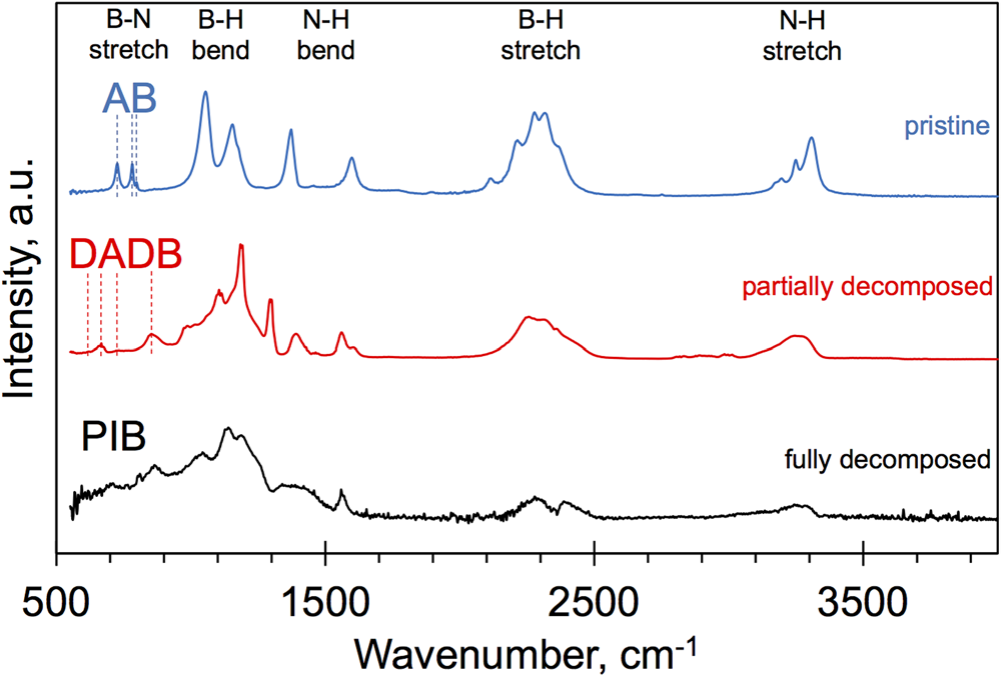
Spectroscopic precursor characterization. FTIR spectra of pristine AB (room temperature), partially decomposed AB (maximum N_x_B_y_ species flow; T_AB_ = 90 °C, 90 min) and after full decomposition (low N_x_B_y_ species flow; T_AB_ = 90 °C, 150 min). Due to the difference in B-N bonding of the AB and DADB molecules, the B-N stretch region can be used to identify AB and DADB phases.

To further confirm the phase change of AB during heating (T_AB_ = 90 °C) we recorded Fourier transform infrared spectra (FTIR) of the precursor at different stages of its decomposition: (i) pristine precursor, (ii) at the maximum flow rate of volatile species (partially decomposed), and (iii) after full decomposition (Fig. 3). The FTIR features can be readily assigned to different modes^43^. Fundamentally different bonding between B-N in AB and DADB means that these can be distinguished from the B-N stretch mode in the 600 cm^−1^–900 cm^−1^ region^43^. AB has three characteristic peaks at 726 cm^−1^, 782 cm^−1^ and 798 cm^−1^, while DADB has two wider separated main peaks at ~667 cm^−1^, ~853 cm^−1^ and two smaller peaks^43^. Notably, AB is no longer detected when the release of N and B-species reaches a maximum, only peaks for DADB can be observed. After complete decomposition of the precursor, the intensity of B-H and N-H modes becomes very low and a broad feature appears in the 600–1500 cm^−1^ region (Fig. 3). This spectrum matches well with polyiminoborane (PIB), reported by previous studies^43^ and it is the solid residue that remains in the precursor chamber after full AB decomposition.

From the exemplar profiles at T_AB_ = 90 °C (Fig. 2a–f) it is evident that the release of N-species is dominated by ammonia during the AB decomposition phase (~80 %), but after the conversion to DADB (~90 min), the majority originates from monomeric aminoborane (~73 %). Various chemical species contribute to the presence of B atoms throughout the measurement: initially diborane and then monomeric aminoborane respectively. These results are summarised in Supplementary Table 2.

### Choice of the precursor temperature for h-BN synthesis

**Figure 4:**
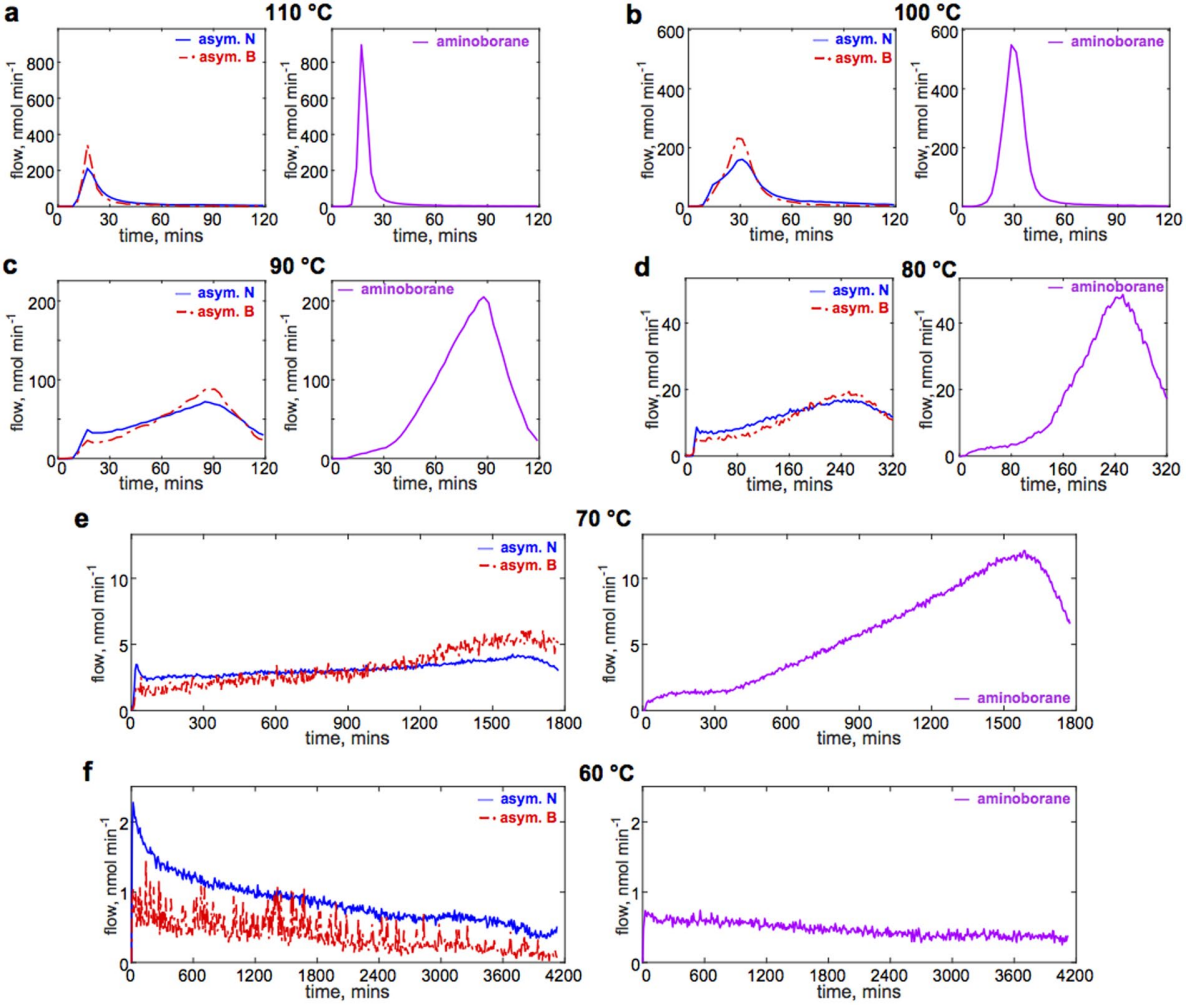
Temporal profiles of ammonia borane decomposition at various precursor temperatures (TAB). (**a**–**f**) Profiles of molar flows of N and B from asymmetric species (left) and of N (or B) from monomeric aminoborane (right) in the temperature range between T_AB_: 60 – 110 °C. For temperatures above 60 °C an apparent change in precursor behaviour can be observed corresponding to the solid-state phase change from AB to DADB (overlapped for 110 °C), as discussed in main text. At lower temperatures the peak is significantly delayed, while the flux of N_x_B_y_-containing species is also lower. At 60 °C simple decomposition and decay occurs. Flows are scaled per 1 mg of AB precursor. The first 5 minutes account for pre-heating the container to 50 °C, followed by rapid heating to the set temperature (methods).

A range of heating conditions for AB have been reported (T_AB_: 60 °C − 130 °C), where often the choice resulted from observations of h-BN deposits after the synthesis. Using the approach proposed here, based on time-dependent measurement of chemical species, it is now possible to systematically assess the suitability of different heating regimes for the tailored production of h-BN. For clarity in further discussion, due to the chemical similarities we distinguish the contribution to the total molar flows of N and B atoms from the “asymmetric” molecules (BH_3_, B_2_H_6_, B_3_H_x_, NH_3_, NB_2_H_x_, B≠N) and also a “symmetric” molecule, aminoborane (the other, heavier symmetric molecules, N_2_B_2_H_x_, N_3_B_3_H_x_, are ignored because of their negligible contribution). The data analysis steps are discussed in the methods section. Figure 4 shows the temporal profiles of total flows of N and B atoms from the asymmetric species and aminoborane for T_AB_: 60 °C − 110 °C: showing the rapid profiles at higher temperatures first followed by the slower profiles. Above 60 °C the profiles exhibit three stages: (i) an induction period where the flows of N and B are nearly constant, followed by a (ii) sigmoidal increase in the concentration of the products (as predicted by the “nucleation and growth” mechanism^30^ for AB polymerisation), followed by (iii) a rapid decay in the concentration. These measurements match the explanation of the solid-state phase change to DADB from previous studies^42^. As would be expected, different time scales and different magnitudes of N, B fluxes are observed at different precursor temperatures (T_AB_). At higher temperatures, the reaction is faster and produces significantly more N_x_B_y_-containing species. For example, at T_AB_ = 70 °C (Fig. 4e), the induction period can be observed up to 400 minutes with the total flow of around 4 nmol min^−1^ for B atoms (similar for N), while at T_AB_ = 90 °C (Fig. 4c) the initial phase shortens to 30 minutes and produces about 50 nmol min^−1^ flow of B atoms. As such, the precursor is exhausted much quicker at higher temperatures (T_AB_). Importantly, the ratio of flows of N to B is not consistent throughout the measurement, which is discussed later in the text.

**Figure 5:**
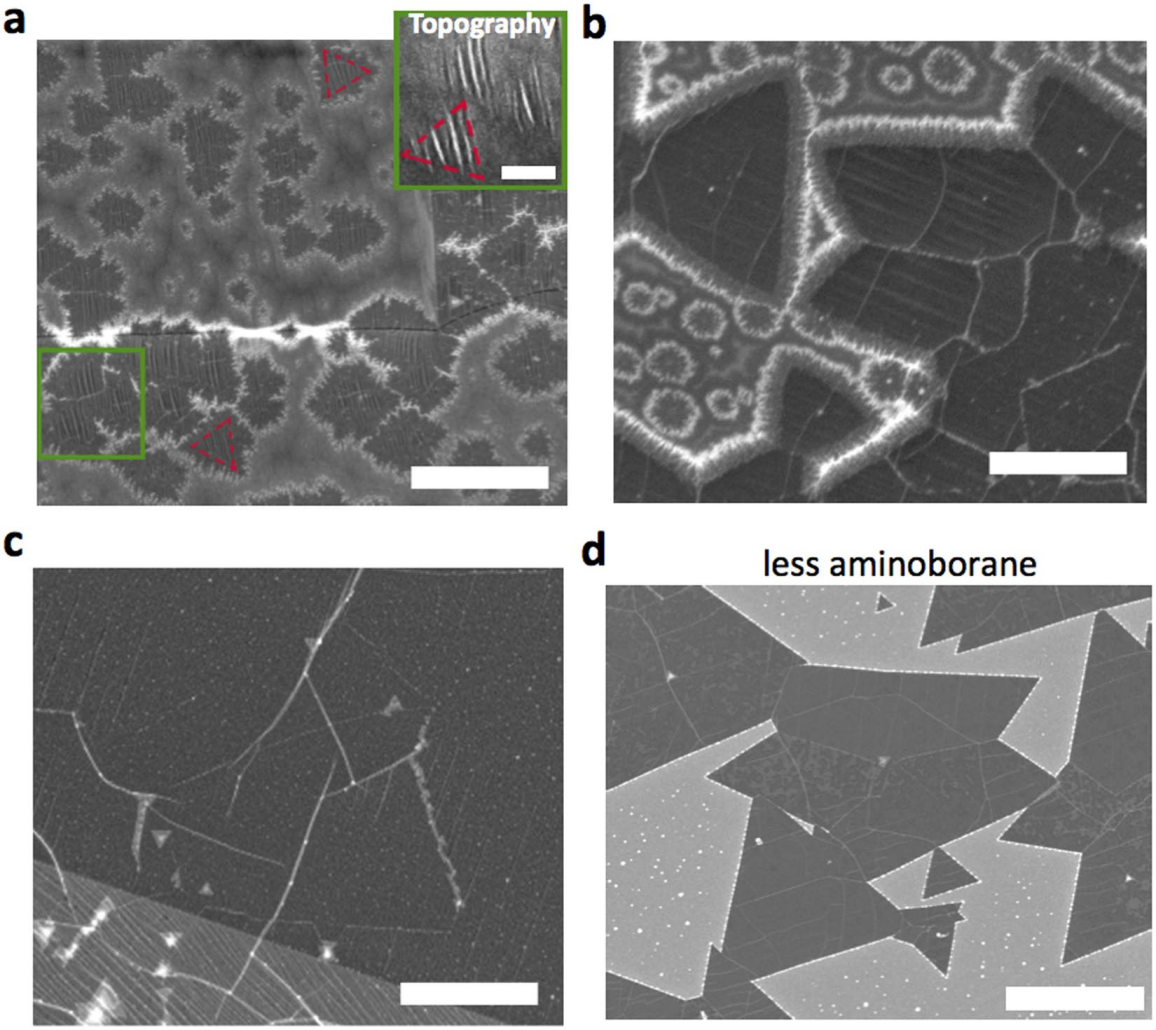
Monomeric aminoborane influence on the morphology of h-BN deposits. (**a**–**c**) Deposits produced with high aminoborane fraction (~55 % of the total flow) at different stages of the growth: after nucleation; with partial and full coverage. Crystalline triangular domains are surrounded with a “fuzzy”, likely amorphous deposit that does not grow in size as the triangular domains. Additionally, separate circular “fuzzy” deposits are observed. The full-coverage h-BN film does not contain the amorphous regions after triangular domains coalesce. (**d**) h-BN domains produced with a lower aminoborane fraction of the total volatile species flow (~30 %). White particles arise from contaminations on the Cu surface. Scale bars (**a**–**c**) 2 μm; inset: 0.5 μm; (**d**) 10 μm.

Notably, the contribution of aminoborane is very significant during the peak in the flow of volatile species for T_AB_ > 70 °C. A number of previous reports^44–47^ observed small circular deposits around triangular h-BN domains, especially with APCVD^44^. These deposits were hypothetically attributed to monomeric aminoborane and its tendency to form oligomers^35,45^. Akin to amorphous C contamination if, for example, ethylene and methane precursors are used simultaneously for the production of graphene. In order to verify this assertion, we performed synthesis at the peak of the flow of volatile species (T_AB_ = 90 °C, 90 min), where the monomeric aminoborane flow accounted for about ~55 % of the total molar flow. The furnace temperature was kept at 1070 °C for this and all other synthesis experiments (methods). We observed circular, “fuzzy” deposits at these conditions (Fig. 5a,b). The deposits either surrounded triangular h-BN domains or were evenly distributed on the surface of Cu. However, they did not significantly grow in size or interfere with the formation of an h-BN film (Fig. 5c). Nevertheless, further studies are needed to examine if these amorphous deposits can cause increased number of defects in the film. When the experiment was performed at lower precursor temperatures (T_AB_ = 60 °C) or before the maximum decomposition rate was reached, such that monomeric aminoborane flow accounted for < 30 % of the total molar flow, only crystalline triangular deposits were observed (Fig. 5d). These observations suggest that monomeric aminoborane is not desirable and thus heating regimes and times should be selected to reduce its presence.

The profile at T_AB_ = 60 °C does not exhibit the precursor phase change and simply decays over the course of the measurement. Analogously to graphene, the flow of any h-BN precursor should be sufficiently constant or increasing to avoid reaching a self-limiting growth regime and the inability to achieve full coverage at larger scale^48^. From the measurements in Fig. 4 it can be noted that the AB decomposition profiles do not have a sufficiently long region of constant volatile species flows. In comparison, for graphene synthesis on Cu, experiments were run for up to 48 hours^49^. As such, none of the precursor decomposition profiles is optimal for the tailored h-BN synthesis; however, the profiles T_AB_ ≤ 70 °C are preferable and were studied further.

### Sequential synthesis experiments at a constant precursor temperature

**Figure 6:**
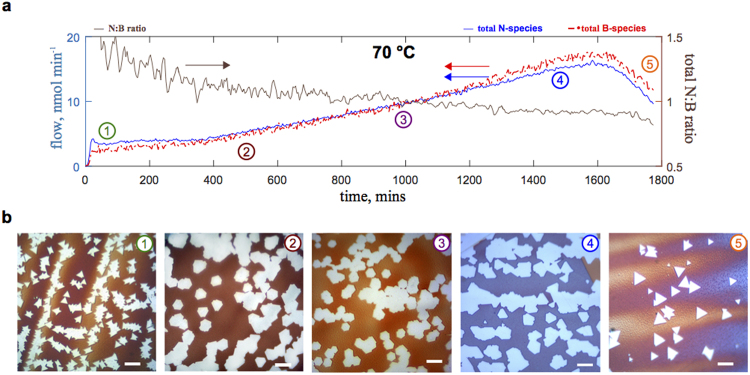
Precursor decomposition profile and selective h-BN synthesis at 70 °C. (**a**) A profile for the total (symmetric and asymmetric) N and B species flows and their ratio are given for the 70 °C precursor temperature (scaled per 1 mg of AB precursor). Notably, there is a variation in the ratio of N and B flows throughout the measurement. (**b**) Exemplar optical images of h-BN domains (white regions) on oxidised (post-synthesis) Cu corresponding to evenly divided time intervals between 0 and 1800 minutes (as marked: 1–5). Initially only triangular domains are observed, followed by domains with various shapes: triangles, truncated triangles, hexagons or mixtures of shapes. To counter the variation in the total molar flow of N and B species, experiments were performed with different synthesis times as to obtain individual h-BN domains (30 min, 20 min, 20 mins, 15 min, 15 min). The growth temperature was 1070 °C. All the scale bars in (**b**) are 10 μm.

From the time-dependent precursor decomposition measurements (Fig. 4a–f), it was found that not only the molar N, B flows varied, their ratio also changed with time, which has not been reported before. The sum of N, B flows (both asymmetric and symmetric) and their ratio is shown in Fig. 6a. The concentration of B was initially lower than N in the gas atmosphere, which inverted (B > N) during the decomposition maximum (and is expected to revert to B < N in the final decomposition stage in line with the shorter profiles in Fig. 4a–d). The cause of this behaviour is unclear, but could be a result the formation of structurally different N_x_B_y_-containing species during different precursor phases. Most importantly, this finding is expected to have a profound effect on the formation of h-BN. It was established in previous studies^50^ that the shape of h-BN domains strongly depends on the ratio of N:B atoms. For example, when N > B, nitrogen edge termination occurs resulting in triangular shape, however, when N = B, hexagonal shapes are formed. An entirely different and more difficult question is what factors control the N:B ratio in the vicinity of growing h-BN domains. Several reasons have been proposed: different N, B dissolution rates^51^, substrate contamination or roughness^20^, different stability of the h-BN edge^52^, imbalance due to an external chemical, *e.g.*, N_2_ carrier gas^50^. Our *in situ* studies now revealed that the AB precursor itself cannot be considered a source of a constant ratio of N to B, even though its chemical formula has a 1:1 stoichiometry.

To understand how different regions of the varying AB decomposition profile (Fig. 6a) affect h-BN growth we performed systematic CVD synthesis experiments from a single precursor loading at different times. The precursor was heated to T_AB_ = 70 °C and the Cu substrate was exposed to the released volatile vapours sampling a period of 30 hours (T_synthesis_ = 1070 °C, 15–30 minutes, methods). The resulting h-BN deposits are shown in Fig. 6b. Only in the first experiment triangular domains were obtained; where the ratio of N:B was sufficiently greater than 1. Notably, AB phase change started soon after this experiment, evident by an increase in the total volatile species release. At later times (experiments 2–5, Fig. 6a,b), a variety of shapes appeared: truncated triangles, hexagons and star-like shapes. Additionally, the synthesis time had to be progressively changed to obtain individual domains and account for the varying release of volatile species.

Without the possibility to control the shape of h-BN domains it is difficult to control domain stitching, leading to inferior film crystallinity. Because of the variation in shape morphology and the level of coverage, methods need to be developed that result in a constant flux of volatile N_x_B_y_-containing species.

### Precursor regulation with varying temperature

**Figure 7:**
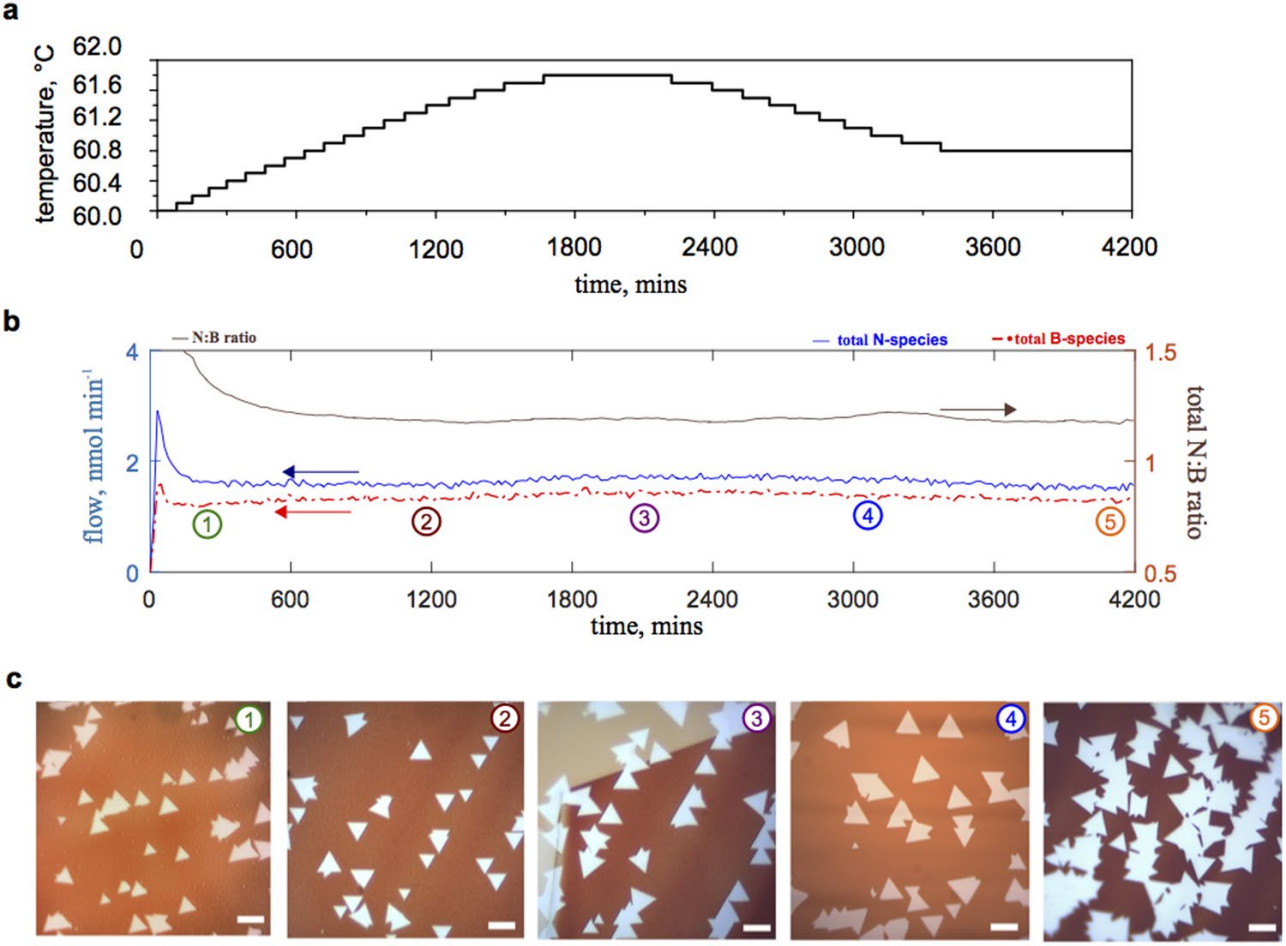
Time-dependent AB decomposition control with temperature regulation. (**a**) The temperature control chart; starting at 60 °C and running for 70 hours. (**b**–**d**) The corresponding N and B total flow profiles and the N:B ratio. (**c**) Optical images of h-BN domains on oxidized Cu showing that the size and shape did not change significantly in the duration of the profile over 70 hours. The synthesis time was also constant between the experiments (30 min; T_synthesis_ = 1070 °C). The observation matched well with the measurement. All the scale bars in (**c**) are 10 μm.

We devised a strategy to achieve constant flows of N and B atoms by varying the precursor temperature with time; which was optimised empirically from a series of measurements and is based on the constant T_AB_ = 60 °C decomposition profile (Fig. 4f). As shown in Fig. 7a, the precursor temperature is slowly increased to counter the decay in the concentration of volatile N_x_B_y_-containing species. The initial ramping rate is very low, around 0.06 °C per hour. Nevertheless, increasing the precursor temperature by only 2 °C after ~1500 minutes started AB phase change to DADB. To prevent a significant increase in the decomposition product formation, the precursor was controllably cooled down. Again, at a very low rate of approximately 0.06 °C per hour. Finally, the precursor temperature did not need significant changes between ~3600–4200 minutes to achieve sufficient consistency. The details of the precursor temperature control points are given in Supplementary Table 3. The resulting total N, B flows (symmetric and asymmetric) and their ratio are shown in Fig. 7b. The ratio of N:B > 1 persisted from the constant T_AB_ = 60 °C profile (Fig. 4f), while the molar flows were made sufficiently constant. As before, a series of synthesis experiments were performed at different points of the developed temperature-regulated profile (T_synthesis_ = 1070 °C, 30 min, methods). In this case, most domains had triangular shape and the size of domains between different experiments was similar (Fig. 7c); without the need to adjust experimental conditions. These measurements also show that temperature deviations of one or few degrees can cause considerable differences in precursor behaviour and thus lead to irreproducibility. As such, it is important to accurately control the precursor in a separate chamber as we suggest (methods), different to the “two-stage furnace” apparatus^20,21,23,27^, where infrared radiation causes significant overheating. Such regulation of AB heating may be a necessary requirement for the controlled growth of h-BN, akin to the need to cool borazine to control its vapour pressure.

Interestingly, in addition to improved shape and size uniformity, these methods allowed us to identify a strategy to re-use the precursor in multiple runs. We found that if partially exhausted precursor was cooled down and then re-heated to the same temperature, the profile of N_x_B_y_-containing species returned to the last value after a short delay (Supplementary Figure 2). This means that the same precursor loading can be used for multiple experiment by simply recording the cumulative time of precursor heating, making experiments more convenient and reducing waste, cost, and time.

### Synthesis optimisation and h-BN characterization

**Figure 8:**
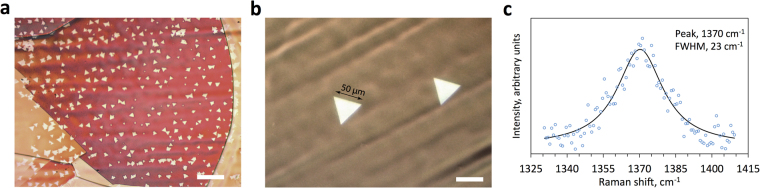
Synthesis optimisation and characterisation of h-BN domains. (**a**,**b**) Comparative images of the improvement in domain size and nucleation density on simply electropolished Cu and electropolished Cu with optimised annealing and synthesis parameters (methods). Domain size is increased by one order of magnitude, while the nucleation density is reduced 3 orders of magnitude as discussed in the main text. (**c**) Raman spectrum of the deposits; exhibiting a significant blue-shift in the E_2g_ peak position (1370 cm^−1^) and a wide FWHM (23 cm^−1^) compared to bulk deposits (1366 cm^−1^, 15 cm^−1^). Consistent with monolayer or very thin h-BN domains^27,45,58^. The scale bars in (**a**,**b**) are 50 μm.

With a uniform, reproducible precursor supply through temperature-controlled decomposition of AB, all the other CVD parameters became much more relevant and strikingly comparable to the established methods for graphene synthesis. It was possible to systematically study and optimise h-BN synthesis towards larger crystal size. We investigated three strategies for lowering the nucleation density in analogy with graphene: 1) electropolishing; 2) oxidation of Cu foil followed by Ar annealing; 3) increasing the H_2_ to precursor flow ratio to attain slower growth rate but reduced nucleation density. Electropolishing significantly reduced the nucleation density, from around 5 × 10^5^ mm^−2^ to 10^3^ mm^−2^, in agreement with previous studies^20,53^ and was used for all samples. Recently, Cu oxidation has been established as the mainstream route towards mm-sized graphene domains with two possible mechanism at play: Cu surface passivation^54^ or scavenging of impurities^55^ that could promote nucleation. However, our experiments showed that this approach was not directly applicable to h-BN because of the formation of elongated and distorted domains as a result (Supplementary Figure 3), suggesting O_2_ interference in the synthesis chemistry. Such influence indicates incompatibility of utilising O_2_ in h-BN growth, while it seems beneficial for graphene. However, it was possible to avoid the formation of the elongated features by further prolonged annealing in a reducing Ar/H_2_ atmosphere for 3 hours. This treatment allowed sufficient time to negate the effects of O_2_ and resulted in appreciable reduction in the h-BN domain nucleation density (10^3^ mm^−2^ to 400 mm^−2^), possibly simply due to enhanced Cu restructuring during oxidative annealing^56^. Finally, the nucleation density was lowered even further by using higher H_2_ flows and longer synthesis times^57,58^. For example, 50 µm domains and nucleation density of ~4 mm^−2^ could be achieved with 500 sccm flow of 2.5% H_2_ in Ar (T_synthesis_ = 1070 °C, 6 hours, methods). Two comparative images of h-BN deposits on simply electropolished Cu and on electropolished Cu with the above optimised synthesis conditions are shown in Fig. 8a,b.

Raman characterization (Fig. 8c) showed that the deposits exhibited a large blue shift in the E_2g_ peak (1370 cm^−1^) and a large full width at half maximum (FWHM) value (23 cm^−1^) compared to bulk or few layer deposits (1366 cm^−1^ and 15 cm^−1^ respectively), suggesting monolayer or very thin h-BN, consistent with previous studies^44,59,60^. Aberration-correct transmission electron microscopy (AC-TEM) was also used to confirm the result by imaging a single layer of h-BN atoms near the domain edge which exhibited hexagonal symmetry (Supplementary Figure 4).

## Discussion

In summary, we show that while ammonia borane is a safe and inexpensive precursor for h-BN synthesis, its behaviour needs to be better understood in order to develop appropriate control methods. *In situ* MS measurements revealed that the stochastic polymeraization and a solid-state phase change of AB are accompanied by the release of multiple volatile N_x_B_y_-containing species upon heating. Moreover, their abundance and the ratio of N:B atoms are not constant with time. Different species can produce different types of deposits, while the time-dependent variation of the synthesis atmosphere leads to poor control of the nucleation density, thickness and shape. We overcome these complexities by simply regulating the precursor temperature with time and monitoring the precursor decomposition with mass spectrometry. A consistent flux of suitable volatile N_x_B_y_-containing species can be achieved and thus, the ability to tailor the type of h-BN deposits. We utilise non-flammable conditions and develop a strategy to re-use the precursor, paving the way towards industrially-applicable production.

## Methods

### Apparatus and controls implementation

Our APCVD system consisted of a stainless steel (SS) gas mixing manifold, precursor evaporation chamber, precursor delivery manifold connected to a fused silica tube and a high temperature furnace (Supplementary Figure 5a). An electrical heater and electrical tape were used to heat the precursor chamber and the SS manifold to 50–110 °C and 90 °C respectively. The set temperature of the heater could be reached quickly with a heating rate of approximately 10 °C min^−1^ and maintained with < 0.1 °C accuracy. An SS needle in the precursor chamber allowed convenient air purging and precursor vapour displacement. It was found that the commercially-available AB precursor released considerable tetrahydrofuran impurity (Supplementary Figure 6) in the early stages of heating, which is used as an intermediate step of the industrial production of AB^61^. For this reason, a by-pass exhaust line connected directly to the evaporation chamber was employed (Supplementary Figure 5a), allowing precursor pre-heating and purging of the system in parallel with annealing of the substrate.

AB polymerisation and foaming is well documented^62^ and presented an additional challenge (Supplementary Figure 5b). This process meant that experiments performed under the same conditions produced appreciably different response due to the random nature of the polymer seed formation and subsequent rapid propagation. To overcome this problem, we mixed AB with an inert filler (BN powder) with weight ratio of 1:10 AB to BN, such that the AB granules were sufficiently separated from each other. Although each granule could still start the polymerisation reaction at random when heated, on average, the time-dependent evolution of N_x_B_y_-containing species was more reproducible and the foaming was suppressed. Supplementary Figure 5c,d schematically summarises the mechanism behind controlling the polymerisation process.

### MS measurements and data analysis

MS measurements were performed on a Hiden Analytical HPR20 quadrupole mass spectrometer with a quartz inert fast sampling capillary. MS pressure was kept at 1E-6 torr. Measurements were performed with a secondary electron multiplier detector at 10^−11^ torr acquisition range and extended dwell times (2000–5000%). Electron energy of 70 V and emission current of 100 μA were used for ionisation. The manifold and the MS sampling capillary were always heated to 90 °C as to obtain comparable flight path conditions for the decomposition products. The travel time of the analytes through the capillary is around 0.3 seconds. The probe was inserted into the fused silica tube, at a position where the furnace would be placed during a CVD experiment. For all measurements, the electrical heater was pre-heated to 50 °C and raised over the evaporation chamber for 5 minutes to avoid any variation in the heating time from ambient temperature; followed by setting the precursor temperature for the measurement^63–66^.

MS intensity profiles are approximately quantified by comparing the base peak of each detected species to the Ar-38 isotope which appears in the ~630 part per million (ppm) concentration^67^. It is understood that this approach could carry an error due to the relative apparatus sensitivity factors (RSF) between the Ar-38 isotope and the measured molecules. It was not possible to obtain correction factors for all of the uncommon species that were detected. Nevertheless, we developed a strategy to approximately correct for the RSF values by first measuring the RSF value of NH_3_ vs. Ar-38 in a calibrated Ar-NH_3_ mixture, which was found it to be only 1.03. After this correction, the absolute error in the asymmetric N-species (dominated by NH_3_ in the early stages of decomposition) was very low, < 3 %. The AB precursor conveniently gave us a way to also correct the scaling of the asymmetric B-species since the ratio of N:B after full AB decomposition is 1:1. Thus, the error in the quantification of the absolute values of the summed flows is estimated to be < 10 %. However, the error in the relative values of the summed flows (*e.g.* N:B ratio) is expected to be even lower, since RSFs divide out. Overall, such MS measurements and quantification are broader and more versatile than what is available with other techniques and is appropriate for the purposes of this study. Data analysis of the 3D MS spectra (m/z, time, intensity) were performed in MATLAB. Briefly, the intensities were scaled to the Ar-38 isotope and multiplied by the total molar gas flow and scaled to 1 mg of the precursor. The background cycle (the cycle before the precursor was heated) was subtracted from all the subsequent cycles allowing to set to zero the background levels of impurity. Any fluctuation in the water level (m/z 18) was corrected for ammonia (m/z 17, m/z 16) and the level of ammonia (m/z 16 scaled to the base peak) was recorded as the final ammonia profile. Similarly, fragmentation patterns presented in the Supplementary Table 1 were used to deconvolute and record profiles of the other N_x_B_y_-containing species. Diborane profile was calculated using the m/z 24 peak, as to avoid a significant overlap with aminoborane; this peak was scaled to the base peak. Diborane contribution was then subtracted from m/z 10–15, 21–28. Monomeric aminoborane, aminodiborane, triborane, borane and borazine profiles were calculated from m/z 27, 43, 37, 13, 80 (scaled to base peaks). For the asymmetric N-species flow, ammonia and aminodiborane flows were added; for the asymmetric B-species flow, 2x diborane, 3x triborane, 2x aminodiborane and 1x borane flows were added together; for the symmetric N-(B-) species flow only the monomeric aminodiborane flow was used due to negligible borazine concentration.

### h-BN synthesis

Cu foil (Alfa Aesar, 99.8 % purity, 25 µm thick) was washed in acetone, followed by electropolishing in 85 % phosphoric acid at 1.8 V for 15 minutes and then annealed in 2.5 % H_2_ in Ar mixture for 30 minutes. The growth temperature was 1070 °C for all CVD experiments. Basic h-BN domains were synthesised with 350 flow of 2.5 % H_2_ in Ar and 110 mg of a mixture of AB/BN (1:10) powder ; at specified precursor temperatures and precursor decomposition times. For the larger, optimised h-BN domain growth, electropolished Cu was oxidized for 60 seconds at 200 °C, followed by 30 min annealing in pure Ar and 3 hour annealing in a 2.5 % H_2_ in Ar mixture. The synthesis was then performed with 500 sccm flow of Ar/H_2_ for 6 hours while the precursor was heated according to the profile in Fig. 7a. The full temperature control chart is given in Supplementary Table 3.

### Characterization

For optical microscopy, the samples were heated to 200 °C for 30 seconds to oxidize the uncovered Cu surface, which changed its colour. Images were recorded on a Yenway-CX40 microscope.

Scanning electron microscope (SEM) images of the samples were recorded using a Carl Zeiss Merlin SEM operating at 5 kV accelerating voltage and 1 nA probe current.

The h-BN transfer was performed using the standard polymer-assisted technique described elsewhere^50^. Raman spectra were recorded on a Horiba LabRAM Aramis microscope equipped with a 532 nm laser. FTIR spectra were recorded on Bio-Rad Varian Excalibur FTS 3500 spectrometer. AFM scans were performed in AC mode on an Oxford Instruments Asylum Research Cypher AFM. An AC160TS probe was used from Olympus Probes. AC-TEM was performed on a double aberration-corrected JEOL JEM-2200MCO microscope at 80 kV.

## Electronic supplementary material

Supplementary Information
