# Publisher Correction: Time dependent decomposition of ammonia borane for the controlled production of 2D hexagonal boron nitride

**DOI:** 10.1038/s41598-017-16514-y

**Published:** 2017-11-22

**Authors:** Vitaliy Babenko, George Lane, Antal A. Koos, Adrian T. Murdock, Karwei So, Jude Britton, Seyyed Shayan Meysami, Jonathan Moffat, Nicole Grobert

**Affiliations:** 1 Department of Materials, University of Oxford, Oxford, OX1 3PH UK; 2 Oxford Instruments Asylum Research, High Wycombe, HP12 3SE UK; 3 Williams Advanced Engineering, Grove, Oxfordshire, OX12 0DQ UK; 4 Present Address: Centre for Advanced Photonics and Electronics, University of Cambridge, 9 JJ Thomson Ave, Cambridge, CB3 0FA UK; 5 Present Address: Department of Chemistry, University of Cambridge, Lensfield Rd, Cambridge, CB2 1EW UK; 6 Present Address: Nanostructures Department, Institute of Technical Physics and Materials Science, Centre for Energy Research, PO Box 49, H-1525 Budapest, Hungary; 7 Present Address: CSIRO Manufacturing, P.O. Box 218, Bradfield Road, Lindfield, New South Wales 2070 Australia; 8 Present Address: Renishaw New Mills, Wotton-under-Edge, Gloucestershire, GL12 8JR UK

*Scientific Reports* **7**:14297; doi:10.1038/s41598-017-14663-8; Article published online 30 October 2017

In the original version of this Article, the ORCID ID for Nicole Grobert was omitted. This has now been corrected in the HTML and PDF versions of the Article.

