# Supplementary material for: Time dependent decomposition of ammonia borane for the controlled production of 2D hexagonal boron nitride
Source: Sci Rep. 2017 Oct 30;7:14297. doi: 10.1038/s41598-017-14663-8 (PMC5662770; doi:10.1038/s41598-017-14663-8)
Supplement: Supplementary file 1 — Supplementary Information [file 41598_2017_14663_MOESM1_ESM.pdf]

# Supplementary Information: time dependent decomposition of ammonia borane for the controlled production of 2D hexagonal boron nitride

Vitaliy Babenko[1†], George Lane[1†], Antal A. Koos[1††], Adrian T. Murdock[1†††], Karwei So[1], Jude Britton[1], Seyyed Shayan Meysami[1], Jonathan Moffat[2], Nicole Grobert[1,3*]

[1] Department of Materials, University of Oxford, Oxford OX1 3PH, UK.
[2] Oxford Instruments Asylum Research, High Wycombe, HP12 3SE, UK.
[3] Williams Advanced Engineering, Grove, Oxfordshire, OX12 0DQ, UK.

[†] Present address: Centre for Advanced Photonics and Electronics, University of Cambridge, 9 JJ Thomson Ave, Cambridge, CB3 0FA, UK.
[††] Present address: Nanostructures Department, Institute of Technical Physics and Materials Science, Centre for Energy Research, PO Box 49, H-1525 Budapest, Hungary.
[†††]Present address: CSIRO Manufacturing, P.O. Box 218, Bradfield Road, Lindfield, New South Wales 2070, Australia

[*] E-mail: nicole.grobert@materials.ox.ac.uk

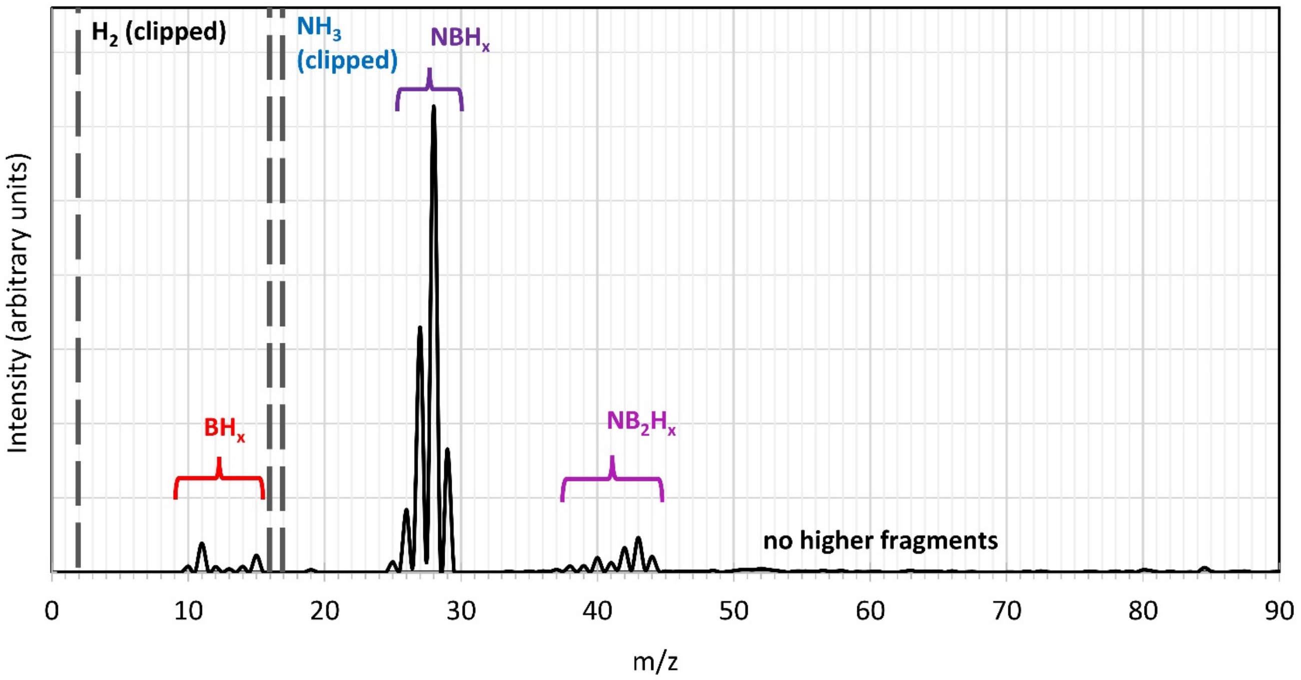


**Supplementary Figure 1**: Mass spectra of AB decomposition when heated in $H_2$-$NH_3$ atmosphere allowing to deconvolute the $B_xH_y$ species.

| $BH_3$ | |
|---|---|
| m/z | intensity |
| 10 | 0.115 |
| 11 | 0.495 |
| 12 | 0.152 |
| 13 | 0.140 |
| 14 | 0.098 |

| $B_2H_6$ (ref 1) | |
|---|---|
| 10 | 0.013 |
| 11 | 0.059 |
| 12 | 0.038 |
| 13 | 0.051 |
| 14 | 0.001 |
| 15 | 0.000 |
| 21 | 0.004 |
| 22 | 0.023 |
| 23 | 0.095 |
| 24 | 0.186 |
| 25 | 0.118 |
| 26 | 0.208 |
| 27 | 0.203 |
| 28 | 0.001 |

| $NH_3$ | |
|---|---|
| 14 | 0.012 |
| 15 | 0.039 |
| 16 | 0.421 |
| 17 | 0.526 |
| 18 | 0.002 |

| $BNH_4$ | |
|---|---|
| m/z | intensity |
| 10 | 0.006 |
| 11 | 0.030 |
| 12 | 0.005 |
| 13 | 0.006 |
| 25 | 0.010 |
| 26 | 0.064 |
| 27 | 0.252 |
| 28 | 0.499 |
| 29 | 0.129 |

| $B_3H_x$ | |
|---|---|
| 36 | 0.024 |
| 37 | 0.060 |
| 38 | 0.137 |
| 39 | 0.208 |
| 40 | 0.162 |
| 41 | 0.204 |
| 42 | 0.205 |

| $B_2NH_x$ | |
|---|---|
| 37 | 0.021 |
| 38 | 0.053 |
| 39 | 0.056 |
| 40 | 0.127 |
| 41 | 0.083 |
| 42 | 0.216 |
| 43 | 0.306 |
| 44 | 0.138 |

| $B_3N_3H_6$ | |
|---|---|
| m/z | intensity |
| 51 | 0.034 |
| 52 | 0.097 |
| 53 | 0.125 |
| 54 | 0.005 |
| 55 | 0.006 |
| 56 | 0.005 |
| 57 | 0.003 |
| 58 | 0.001 |
| 59 | 0.001 |
| 60 | 0.005 |
| 61 | 0.018 |
| 62 | 0.046 |
| 63 | 0.053 |
| 64 | 0.003 |
| 65 | 0.003 |
| 66 | 0.003 |
| 67 | 0.007 |
| 68 | 0.006 |
| 69 | 0.003 |
| 70 | 0.002 |
| 71 | 0.001 |
| 72 | 0.001 |
| 74 | 0.003 |
| 75 | 0.012 |
| 76 | 0.025 |
| 77 | 0.032 |
| 78 | 0.053 |
| 79 | 0.137 |
| 80 | 0.220 |
| 81 | 0.087 |
| 82 | 0.004 |

**Supplementary Table 1**: m/z intensity profiles of the identified N-, B- species.

| | ammonia | diborane | triborane | aminoborane | aminodiborane | borane | borazine |
|---|---|---|---|---|---|---|---|
| Initial decomposition (AB) | | | | | | | |
| **Contribution to N flow, at. %** | 84% | - | - | 15% | 1% | - | 0% |
| **Contribution to B flow, at. %** | - | 64% | 4% | 28% | 4% | 0% | 0% |
| Peak decomposition (DADB) | | | | | | | |
| **Contribution to N flow, at. %** | 26% | - | - | 73% | 1% | - | 0.3% |
| **Contribution to B flow, at. %** | - | 3% | 19% | 73% | 2% | 2% | 0.3% |

**Supplementary Table 2: Percentage contributions of different chemical species to the B and N molar flows.** Two stages are shown when heated to 90 °C. Initially, simple AB decomposition products (amminora and diborane) dominate. After the conversion to the more mobile DADB phase, monomeric aminoborane release accounts for the highest contribution to both B and N molar flows.

| time, minutes | temperature, °C |
|---|---|
| 0 | 60 |
| 83 | 60.1 |
| 150 | 60.2 |
| 223 | 60.3 |
| 300 | 60.4 |
| 381 | 60.5 |
| 464 | 60.6 |
| 549 | 60.7 |
| 634 | 60.8 |
| 719 | 60.9 |
| 804 | 61 |
| 890 | 61.1 |
| 977 | 61.2 |
| 1066 | 61.3 |
| 1159 | 61.4 |

| time, minutes | temperature, °C |
|---|---|
| 1258 | 61.5 |
| 1367 | 61.6 |
| 1494 | 61.7 |
| 1665 | 61.8 |
| 2216 | 61.7 |
| 2391 | 61.6 |
| 2523 | 61.5 |
| 2639 | 61.4 |
| 2748 | 61.3 |
| 2854 | 61.2 |
| 2962 | 61.1 |
| 3077 | 61 |
| 3207 | 60.9 |
| 3376 | 60.8 |
| 4200 | 60.8 |

**Supplementary Table 3**: temperature vs. time chart for the consistent volatile N-, B- species release from the ammonia borane precursor.

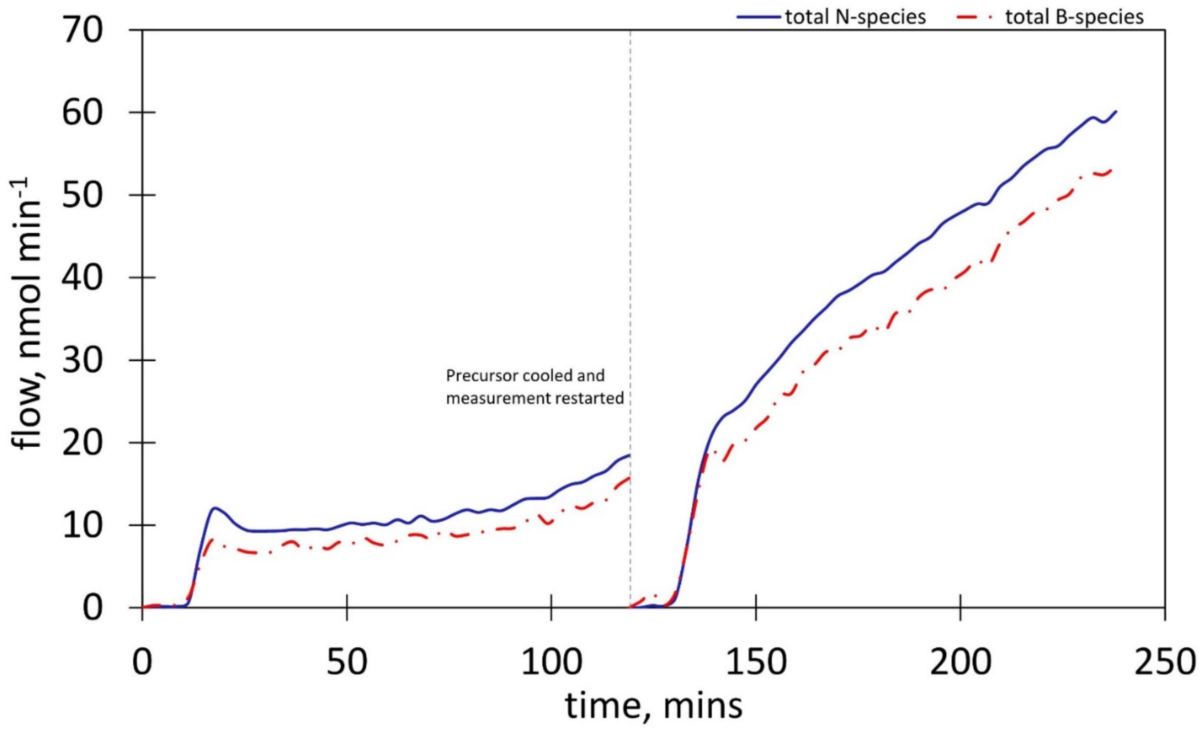


**Supplementary Figure 2**: the total molar flow of N (blue) and B (red) at 80 °C with the precursor cooled and re-heated in two separate measurements. For clarity plotted on a continuous time-scale.

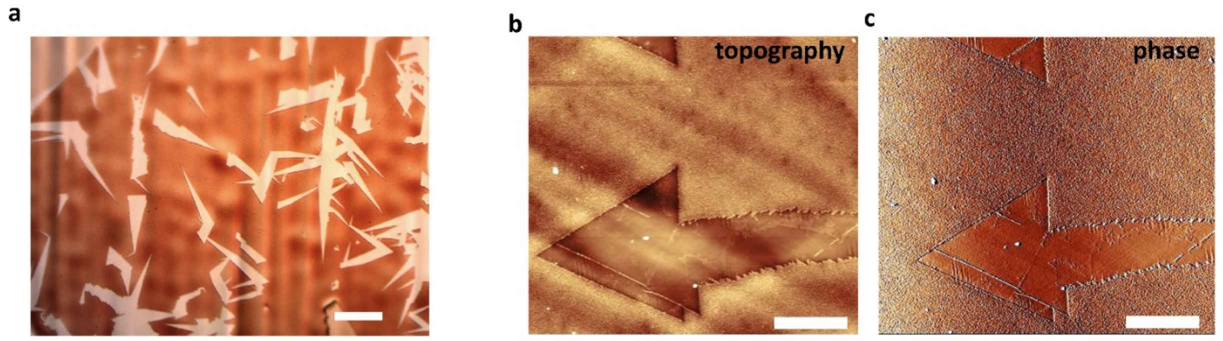


**Supplementary Figure 3**: h-BN synthesis on oxidized Cu foils resulting in elongated domains or dendritic tail growth. a) An optical image of the elongated h-BN deposits. Scale bar 50 µm. b-c) Atomic force microscopy scans of the domains showing topography and phase respectively. Scale bars are 5 µm.

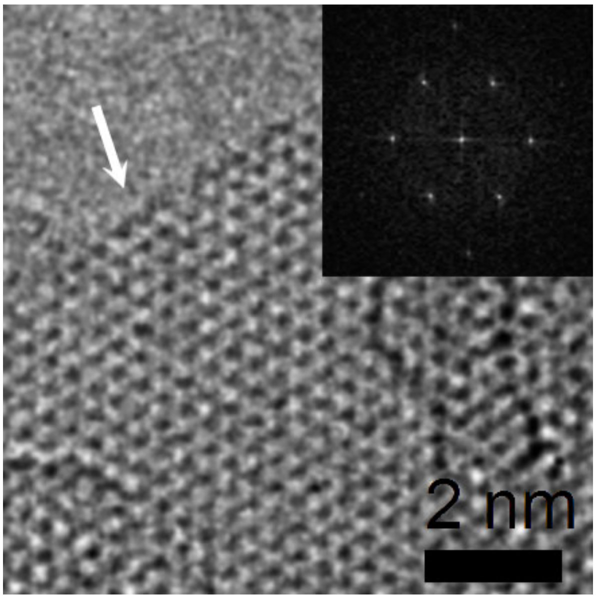


**Supplementary Figure 4**: Aberration-corrected transmission electron microscope image of the edge of h-BN domains (indicated by arrow) showing monolayer nature of the domain. Inset: a Fourier transform pattern of the image, confirming hexagonal symmetry of the BN lattice.

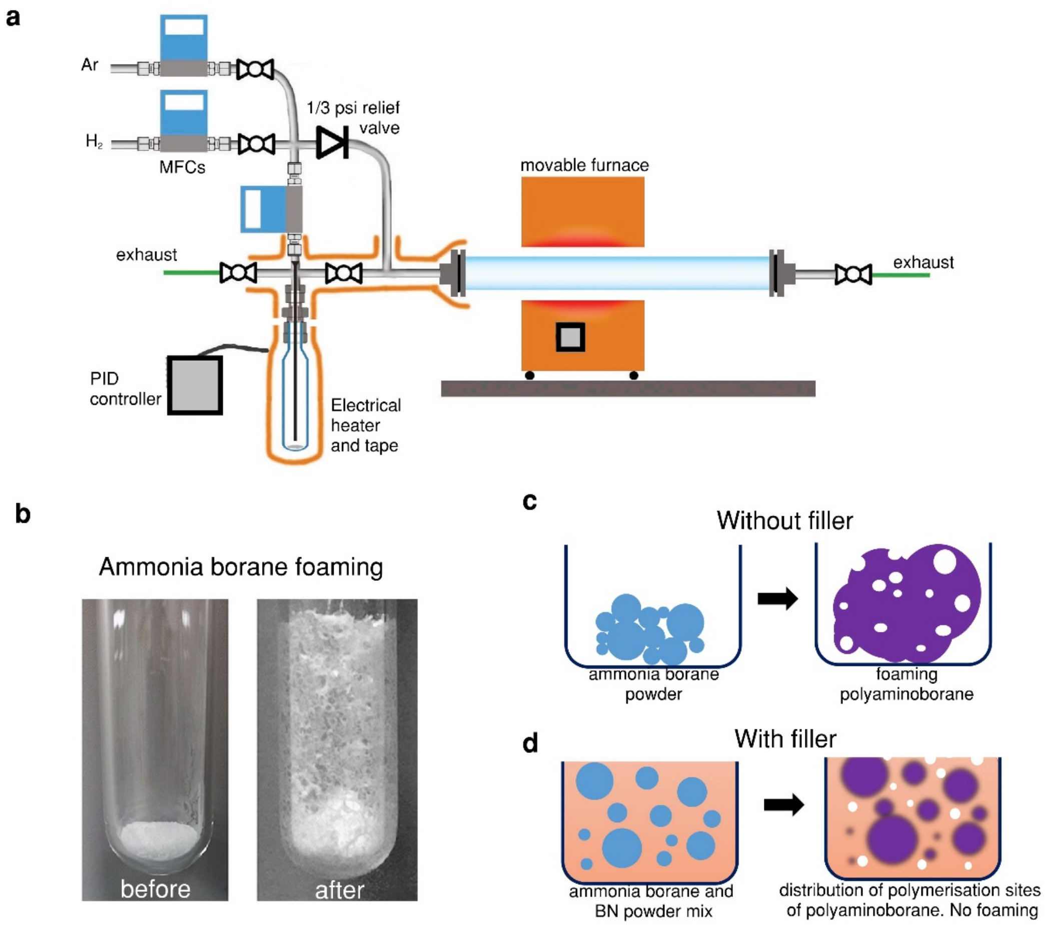


**Supplementary Figure 5**: The CVD system for h-BN synthesis and AB foaming. (a) A schematic of the h-BN CVD system showing its main components. (**b**) Optical images of pristine AB precursor decomposition before and after heating, showing significant foaming and polymerization. However, with the AB/BN powder mix (methods) there was no visible change to the precursor volume after heating. (**c-d**) Diagrams of AB powder polymerisation without and with an inert filler; a distribution of polymerization sites significantly reduces global polymerization and foaming.

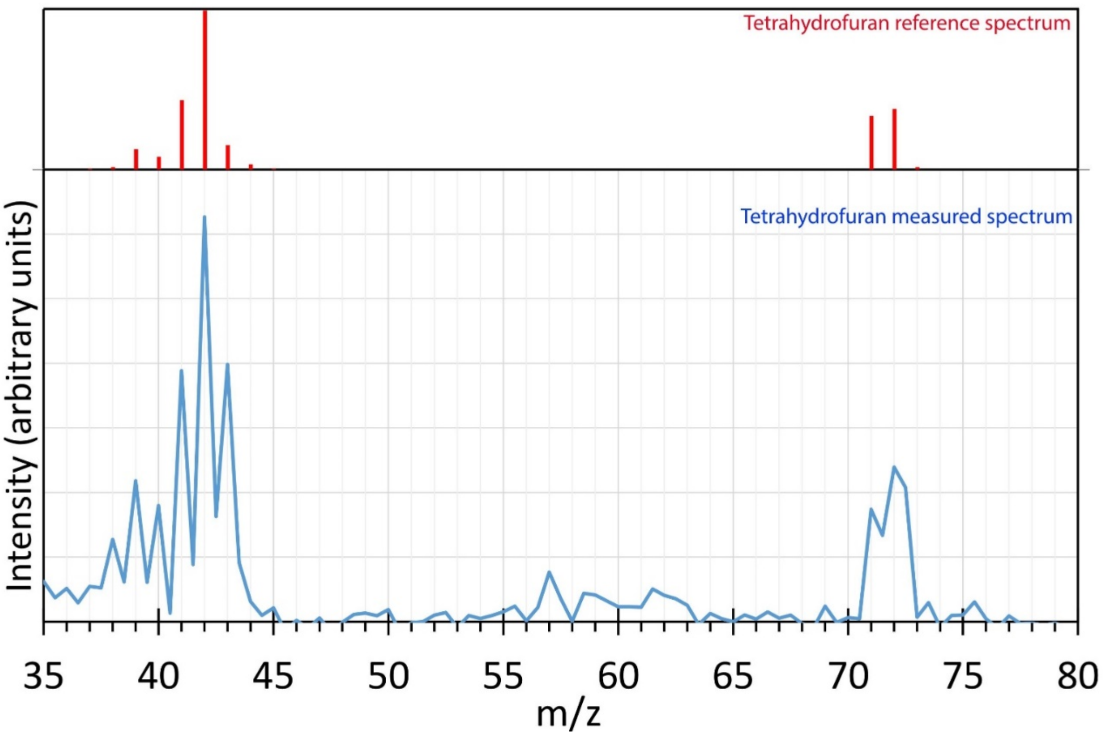


**Supplementary Figure 6**: Tetrahydrofuran impurity detected with MS in the early stages of precursor heating, which quickly disappeared. Reference spectrum is given above[1].

## References


1. Stein, S.E. in NIST Chemistry WebBook, NIST Standard Reference Database Number 69. (eds. P.J. Linstrom & W.G. Mallard) (March 28, 2017).
